# Recurrent Atrial Fibrillation Ablation after Initial Successful Pulmonary Vein Isolation

**DOI:** 10.3390/jcm12227177

**Published:** 2023-11-19

**Authors:** Julian Müller, Karin Nentwich, Artur Berkovitz, Kai Sonne, Olena Kozlova, Sebastian Barth, Alexandru Deacanu, Christian Waechter, Philipp Halbfass, Heiko Lehrmann, Thomas Deneke

**Affiliations:** 1 Clinic for Interventional Electrophysiology, Heart Centre Bad Neustadt, 97616 Bad Neustadt a. d. Saale, Germanyolena.kozlova@campus-nes.de (O.K.); halbfass@gmx.net (P.H.);; 2 Department of Cardiology, Faculty of Medicine, University Heart Center Freiburg-Bad Krozingen, University of Freiburg, 79085 Freiburg im Breisgau, Germany; heiko.lehrmann@uniklinik-freiburg.de; 3 Department of Cardiology and Angiology, Philipps-University Marburg, 35037 Marburg, Germany; christian.waechter@staff.uni-marburg.de

**Keywords:** ablation index, atrial fibrillation, redo procedure, pulmonary vein isolation, high-power short-duration ablation, prognosis, pulmonary vein reconnection

## Abstract

Background: Pulmonary vein isolation (PVI) is an effective treatment option for patients with symptomatic atrial fibrillation (AF). However, the electrical recovery of pulmonary veins (PVs) is the main trigger for AF recurrences. This study investigates the characteristics of patients admitted for redo AF ablation, the PV reconnection rates depending on previous ablation modalities and the impact of different ablation strategies for redo procedures. Methods: Consecutive patients undergoing first redo AF ablation were included. Patients were grouped according to the electrical recovery of at least one PV. The impacts of the technique for first AF ablation on PV reconnection rates and patients with and without PV reconnection were compared. Different ablation strategies for redo procedures were compared and its recurrence rates after a mean follow-up of 25 ± 20 months were investigated. Results: A total of 389 patients (68 ± 10 years; 57% male; 39% paroxysmal AF) underwent a first redo. The median time between the first and redo procedure was 40 ± 39 months. Radiofrequency was used in 278 patients, cryoballoon was used in 85 patients and surgical AF ablation was performed on 26 patients. In total, 325 patients (84%) had at least one PV reconnected, and the mean number of reconnected PVs was 2.0 ± 1.3, with significant differences between ablation approaches (*p* for all = 0.002); this was mainly due to differences in the left inferior PV and right superior PV reconnections. The presence of PV reconnection during redo was not associated with better long-term success compared to completely isolated PVs (67% vs. 67%; log-rank *p* = 0.997). Overall, the different ablation strategies for redos were comparable regarding AF recurrences during follow-up (*p* = 0.079), with the ablation approach having no impact in the case of left atrial low voltage or without. Conclusions: PV reconnections after initial successful PVI are common among all techniques of AF ablation. Long-term rhythm control off antiarrhythmic drugs was possible in 2/3 of all patients after the redo procedure; however, different ablation strategies with extra-PV trigger ablation did not improve long-term success. Patients with recurrent AF after PVI constitute a challenging group of patients.

## 1. Introduction

Atrial fibrillation (AF) is the most common sustained tachyarrhythmia worldwide and is strongly associated with increased morbidity and mortality [1]. Radiofrequency (RF) catheter ablation has become an effective treatment option in symptomatic patients, and the electrical isolation of the pulmonary veins (PVI) is the cornerstone of every AF ablation procedure [2,3]. 

However, recurrent AF after initial successful PVI still occurs in around 1/3 of all patients and often requires repeat ablation [4,5]. The electrical reconnection of one or more PVs is considered a major mechanism of AF recurrence [6,7]. Reconnection is mostly related to non-transmural lesions due to tissue oedema. Nevertheless, especially in patients after initial long-term success, some patients reveal recurrent AF despite silent PVs [8]. In patients with non-PV foci of AF undergoing de novo ablation, several strategies have been proposed, including the targeting of specific sites with low-voltage zones (LVZ) or selected structures, such as the superior vena cava (SVC), left atrium (LA) posterior wall, coronary sinus (CS) or left atrial appendage (LAA) [9,10]. However, for patients with recurrent AF and electrically silent PVs during redo ablation, little is known about the optimal strategy able to achieve long-term success or its implications. 

Prospective lesion durability evaluations are limited and seem to differ between ablation modalities. The FIRE AND ICE trial found that cryoballoon (CB) procedures were associated with fewer reconnected PVs, especially fewer superior PV reconnections compared to radiofrequency (RF) ablations. 

The purpose of this study was to evaluate the long-term durability of PVI with different ablation modalities and its reconnection patterns. Furthermore, the impact of silent PVs with recurrent AF on patients’ long-term outcome after redo procedures was investigated and different ablation strategies for redo procedures were evaluated.

## 2. Methods

### 2.1. Study Population

All patients undergoing redo AF ablation in our department from January 2019 until May 2022 consecutively were included in this study and screened for eligibility. 

This study comprised 3 major stages. Patients undergoing their first redo procedure were analyzed and their PV reconnection pattern was investigated in regard to the ablation technique for the initial AF ablation procedure. In a second step, the long-term follow-ups of patients with and without reconnected PVs were compared. In a third step, different ablation strategies for redo procedures depending on the presence of additional extra-PV ablation targets and (LVZ) were compared. Data were evaluated in a retrospective design based on the previous discretion of the operator. 

Major complications were defined as complications prolonging hospital stay and/or requiring additional intervention/surgery. 

All patients gave written informed consent to the ablation procedure and all pre- and post-ablation diagnostics. The study was carried out according to the principles of the declaration of Helsinki and was approved by the local medical ethics committee of the Heart Centre Bad Neustadt, Germany (approval code: Studie_1_2020).

### 2.2. Atrial Fibrillation Ablation Procedure

Our detailed ablation protocol has been described in detail elsewhere [11]. In brief, mapping was performed using a dedicated three-dimensional electroanatomic mapping system (CARTO 3, Biosense Webster, Diamond Bar, CA, USA) in combination with a decapolar mapping catheter (LASSO; BiosenseWebster, Diamond Bar, CA, USA) or, in selected patients, with a multipolar catheter (PENTARAY; Biosense Webster, Diamond Bar, CA, USA). For AF ablation, a ThermoCool SmartTouch SF (Biosense Webster, Irvine, CA, USA) in combination with the SMARTABLATE generator (Biosense Webster, Irvine, CA, USA) was used. An inter-tag distance of ≤6 mm was aimed for and the target contact force was 10 to 25 g at all ablation sites. AI-guided RF energy was applied (AI 350 for posterior wall ablation, AI 450 for anterior wall ablation). All patients were therapeutically anticoagulated at the time of the ablation procedure. All redo ablations were performed using a radiofrequency energy of 50 W, except for SVC isolations (25 W). 

As a first step, all ablation procedures targeted the re-isolation of reconnected PVs. As a second step, substrate voltage mapping was performed and substrate modification, including left and right atrial flutter ablations (if applicable), was performed. In addition, SVC ablations were performed with prior pacing and assessment for phrenic nerve stimulation before the application of energy at the discretion of the operator. 

In the case of remaining AF after ablation, electric cardioversion was performed and the confirmation of the entry and exit block of all PVs during sinus rhythm was performed. Additional ablation in sinus rhythm for the adequate modification of non-PV triggers and the remaining PV connection was performed when needed. 

In some patients, the dormant conduction of PVs was assessed using adenosine boluses. In all patients with previously documented typical atrial flutter, an ablation of the cavotricuspid isthmus was performed, with confirmation of bidirectional block at the end of the procedure. 

In total, 6 different ablation strategies for redo procedures based upon the presence of a PV reconnection, as well as the operator’s preference, were applied. In total, if a PV reconnection and no LA substrate were present, the segmental re-isolation of all PVs with or without SVC isolation was performed (group 1 and 2). If a further LA substrate was present, modification was performed at the operators’ discretion (group 3: PV re-isolation only without LA substrate modification but LVZ documented; group 4: PV re-isolation and LA substrate modification without additional SVC isolation; group 5: PV re-isolation and LA substrate modification with additional SVC isolation). If no PV reconnection was present, LA substrate modification (if applicable) with additional SVC isolation was performed (group 6). All AF ablation procedures were performed by highly experienced operators, each having performed >1000 AF ablation procedures previously. All operators held an invasive electrophysiology diploma of the European Heart Rhythm Association (EHRA) and the German Cardiac Society (DGK). 

Esophageal endoscopy was performed within the next workday after AF ablation. Endoscopically detected thermal esophageal lesions were defined as previously published by our group [12]. The differentiation was performed based on visual aspects during the initial endoscopy. 

### 2.3. Study Endpoint

The study endpoint was the recurrence of any atrial tachyarrhythmia after a 3-month blanking period. Antiarrhythmic drugs and/or electrical cardioversion were used to treat Arrhythmia recurrences within the blanking period. Atrial arrhythmia recurrences after the blanking period were defined as either an episode of symptoms related to AF or ECG-documented atrial tachycardia. 

### 2.4. Follow-Up

To evaluate the procedural efficacy, multiple (at least two within the first 3 months, then every 3 months after) 24–72-h Holter ECG recordings and clinical evaluation in relation to AF episodes were used. If clinical symptoms potentially related to AF were noted, an in-house follow-up was scheduled and an ECG or 24–72 h Holter ECG was performed. If symptoms were present but no ECG document at that time was available, further ECG monitoring including telemonitoring ECG devices was used. The follow-up protocol was the same irrespective of operator, ablation protocol or strategy. 

### 2.5. Statistical Methods

Quantitative data are presented as mean ± standard error of mean (SEM), median and interquartile range (IQR), and ranges depending on the distribution of the data were compared using the Student’s *t* test for normally distributed data or the Mann–Whitney *U* test for nonparametric data. Deviations from a Gaussian distribution were tested using the Kolmogorov–Smirnov test. Spearman’s rank correlation for non-parametric data was used to test univariate correlations. Qualitative data are presented as absolute and relative frequencies, and compared using the Chi^2^ test or the Fisher’s exact test, as appropriate. 

The following analyses were applied stepwise to evaluate the prognostic impact of predefined variables on the study endpoints: Kaplan–Meier curves were calculated with log-rank testing for statistical significance. Uni-variable hazard ratios (HR) are given together with 95% confidence intervals. Multi-variable Cox regression models with stable sinus rhythm after follow-up as the dependent variable were developed using the “forward selection” option. Multi-variable models were adjusted using both univariably statistically significant variables and clinically relevant variables. The result of a statistical test was considered significant for *p* < 0.05; *p* values ≤ 0.1 were defined as a statistical trend. SAS, release 9.4 (SAS Institute Inc., Cary, NC, USA) and SPSS (Version 25, IBM Armonk, New York, NY, USA) were used for statistics. 

## 3. Results

### 3.1. Baseline Characteristics 

Between January 2019 until May 2022, 584 consecutive patients undergoing a redo AF ablation procedure at our institution were identified out of a total of 1756 AF procedures. In total, 389 procedures were first redos (and were included for further analyses), and 139 were second, 41 third, 7 forth and 1 fifth redo procedures. Among the patients undergoing first redo procedures, AF was paroxysmal in 150 (39%). Most patients were males (57%), and their mean age was 68 years. The mean follow-up time was 25 ± 20 months (see Table 1).

The mean time between first ablation procedure and redo was 40 ± 39 months. In 178 patients, the initial ablation approach was RF LPLD with 30–35 W (mean time between first and redo 42 ± 30 months); in 96 patients, RF HPSD with 50 W (11 ± 6 months); in 4 patients, RF vHPvSD 90 W (9 ± 4 months); in 31 patients, CB 1st generation (122 ± 40 months); in 17 patients, CB 2nd generation (62 ± 24 months); in 37 patients, CB 3rd generation (16 ± 11 months), and in 26 patients, surgical AF ablation (48 ± 44 months) (*p* for all <0.001). 

In all patients, the initial strategy was typically antral PV isolation and, depending on the respective ablation approach, LVZ substrate modification, depending on the operators’ preference (in total, 60 patients: 38 box lesions, 40 anterior lines, 31 roof lines). Additionally, in 45 patients, a cavotricuspidal isthmus ablation due to documented typical flutter was performed during initial ablation. 

### 3.2. Electrophysiological Findings during Redo and PV Reconnection Rates

Of the 389 patients admitted for first redo, 181 (46%) presented with sinus rhythm, 127 (33%) with AF and 81 (21%) with atrial flutter. The reconnection of at least one PV was found in 325 patients (84%). Reconnections of the left superior, left inferior, right superior and right inferior PV were found in 168 (43%), 134 (35%), 229 (59%) and 238 (61%) patients, respectively (Table 2). In four patients, a left common trunk was present, which was reconnected in all cases. The probability of at least one reconnected PV was highest in patients with sinus rhythm at the beginning of the redo procedure (87% with at least one reconnected PV) and decreased with AF to 84% and with macro re-entrant atrial tachycardia to 75% (*p* = 0.059). The reconnection rates between different ablation techniques are presented in Table 3. The reconnection rates of the right PVs were significantly different (*p* = 0.006 and *p* = 0.051, respectively). The mean number of reconnected veins was 2.0 ± 1.3, with significant differences between ablation approaches (RF LPSD 1.9 ± 1.2; RF HPSD 1.8 ± 1.3; RF vHPvSD 2.5 ± 0.6; CB 1st gen 2.5 ± 1.0; CB 2nd gen 2.5 ± 1.5; CB 3rd gen 2.3 ± 1.3; surgical 1.2 ± 1.6; *p* for all = 0.002). Comparing the RF point according to the point procedures with CB-based procedures, we found significantly increased PV reconnections among CB procedures (1.9 ± 1.3 vs. 2.4 ± 1.2; *p* = 0.002) among all PVs (LSPV 33% vs. 54%; *p* = 0.001; LIPV 28% vs. 45%; *p* = 0.001; RSPV 45% vs. 62%; *p* = 0.003; RIPV 50% vs. 64%; *p* = 0.016).

An additional low-voltage area was present in 227 patients (58%); this was allocated to the anterior wall in 169 (43%), to the roof in 125 (32%), to the posterior wall in 136 (35%) and to the septum in 62 (16%) patients. Additional non-PV ablations were performed in 310 patients (79%), as follows: posterior wall (29%), roof (26%), anterior wall (38%), septum (8%), SVC (47%) and CTI (10%) (Table 2). 

Major complications occurred in nine patients (2.3%), including one pericardial tamponade, two strokes (with prior exclusion of LAA thrombi with TOE), two air embolism with transient ST segment elevations, one RA pacemaker lead dislocation and three groin complications requiring surgery. Minor complications occurred in an additional eight patients (2.1%), including groin hematoma with conservative treatment in eight cases. Endoscopically detected esophageal lesions (EDEL) occurred in six patients, including category 1 EDEL in two and category 2 EDEL in four patients. 

### 3.3. Strategies for Redo Procedures

**Figure 1:**
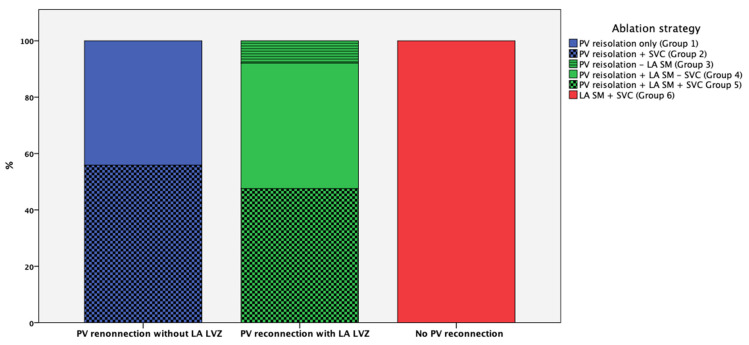
Different re-ablation strategies according to the presence or absence of PV reconnection and LA LVZ. Group 1: The re-isolation of all reconnected PVs “only” without evidence of LA LVA; Group 2: The re-isolation of all reconnected PVs with additional SVC isolation; Group 3: The re-isolation of all reconnected PVs “only” with evidence of LA LVA; Group 4: The re-isolation of all reconnected PVs with LA substrate modification without additional SVC isolation; Group 5: The re-isolation of all reconnected PVs with LA substrate modification and additional SVC isolation; Group 6: LA substrate modification with additional SVC isolation with presence of electrically silent PVs.

In total, 162 patients (42%) had no LA-low voltage area, and the re-isolation of all PVs “only” was conducted in 71 patients (18%; group 1); meanwhile, in 90 patients (23%; group 2), the re-isolation of PVs with additional SVC isolation was performed. Among those patients with PV reconnections and LA low-voltage areas, 13 patients (3%) received PV re-isolation only without LA substrate modification (group 3), 73 patients (19%) received PV re-isolation and LA substrate modification without additional SVC isolation (group 4), and 78 patients (20%) received additional SVC isolation (group 5). In total, 64 patients (16%) showed no PV reconnection and LA substrate modification, and additional SVC isolation was performed (group 6) (Figure 1). 

**Figure 2:**
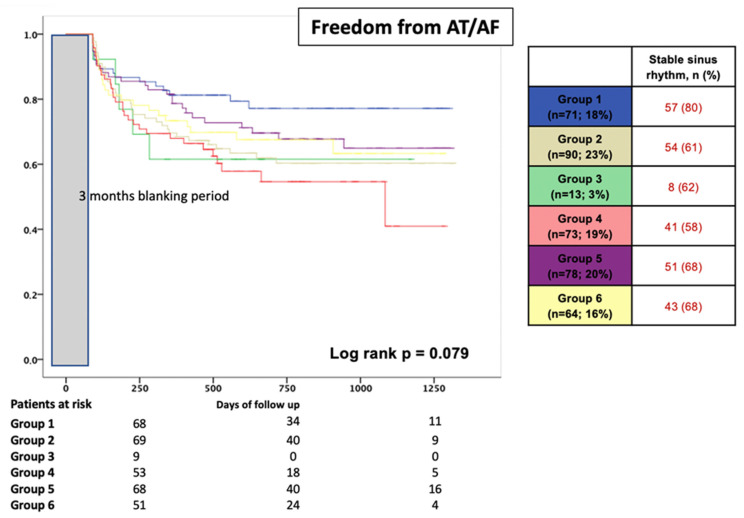
Proportion of patients in stable SR after redo procedure during a mean FU period of 25 ± 20 months, stratified according to the re-ablation strategy. Group 1: The re-isolation of all reconnected PVs “only” without evidence of LA LVA; Group 2: The re-isolation of all reconnected PVs with additional SVC isolation; Group 3: The re-isolation of all reconnected PVs “only” with evidence of LA LVA; Group 4: The re-isolation of all reconnected PVs with LA substrate modification without additional SVC isolation; Group 5: The re-isolation of all reconnected PVs with LA substrate modification and additional SVC isolation; Group 6: LA substrate modification with additional SVC isolation with presence of electrically silent PVs. Grey bar indicated 3 months blanking period after procedure.

The different ablation protocols overall were comparable regarding AF recurrences during follow-up (log-rank *p* = 0.079) (Figure 2). In patients with PV reconnection, the additional empirical isolation of the SVC revealed no prognostic benefit during follow-up (80% without SVC vs. 61% with SVC; *p* = 0.010). In patients with PV reconnection and further LA low-voltage areas, the long-term success rates were also comparable (62% for group 3 vs. 68% for group 5 vs. 58% for group 4; *p* = 0.523) (Figure 2). 

### 3.4. Clinical Outcome after Redo Procedure

After AF ablation, 78 patients (20%) were discharged on antiarrhythmic drugs. After a 3-month blanking period, 332 patients (87%) had stable sinus rhythm. After 1 year, 277 patients (73%) still had stable sinus rhythm off AADs. After a mean follow-up time of 25 ± 20 months, 255 patients (67%) had stable sinus rhythm (21 on AADs). Of those patients with AF/AT recurrence, 52 (41%) had repeat ablation 11 ± 8 months later (Table 4).

### 3.5. Impact of PV Reconnection on Ablation Outcome

When comparing patients with and without PV reconnections at redo, patients with at least on reconnected PV were younger (*p* = 0.021), were predominantly male (*p* = 0.001), were more likely to have paroxysmal AF and had lower CHA2DS2-VASc scores (*p* = 0.001) (Table 1). During the EP study, patients with PV reconnection had fewer LA low-voltage areas (all *p* = 0.001), received fewer extra-PV ablations (all *p* = 0.001, except for SVC), and had consecutive shorter total ablation times (*p* = 0.023) (Table 2). The only independent predictor of no PV reconnection was female gender (OR 2.299, CI 1.148–4.600, *p* = 0.019) in the multi-variable regression analysis.

**Figure 3:**
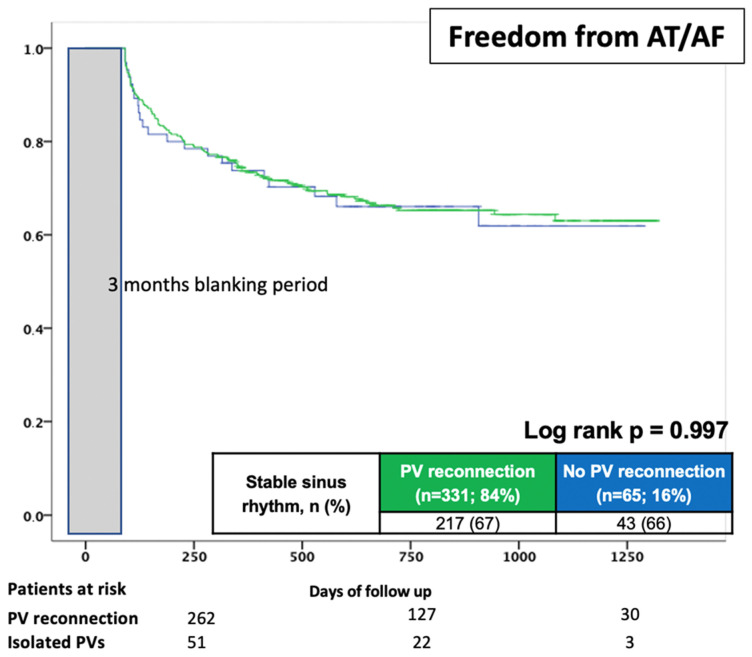
Proportion of patients in stable SR after the redo procedure during a mean FU period of 25 ± 20 months, stratified according to the presence of PV reconnection. Grey bar indicated 3 months blanking period after procedure.

Regarding short and long-term freedom from AF recurrences, patients with and without PV reconnections had comparable rates of sinus rhythm after a mean of 25 ± 20 months; recurrence was 67% among patients with PV connections compared to 67% for patients without (log-rank *p* = 0.997) (Table 4) (Figure 3). 

In a multi-variate analysis of the whole cohort, higher CHADS Vasc Scores (HR 1.281, CI 1.027–1.598, *p* = 0.028) and female gender (HR 2.145, CI 1.725–3.412, *p* = 0.007) were independent predictors of the recurrence of atrial tachycardias during follow-up, but not different ablation approaches (Table 5). To correlate possible confounders among patients with and without LA LVZ, we conducted a multi-variable regression analysis. Here, among age, BMI, the number of reconnected PVs, the type of AF and LA size, only a higher CHA2DS2 VASc Score (HR 1.281, CI 1.027–1.598, *p* = 0.028), AF/AT at the beginning of the procedure (compared to sinus rhythm) (HR 1.656, CI 1.598–2.604, *p* = 0.029) and female gender (HR 2.108, CI 1.615–4.012, *p* = 0.007) were significantly associated with AF recurrence in a multi-variable model, but not additional SVC isolation (Table 6).

In patients with PV reconnection and additional LA LVZ, none of the above-mentioned factors remained associated with AF recurrence, especially not different ablation strategies (Table 7). 

## 4. Discussion

### 4.1. Main Findings

The present study is a large single-center cohort study of first redo AF ablations after different ablation modalities at different ablation centers, investigating the electrophysiological findings and PV reconnection. The results indicate that PV reconnection is still an important driver for recurrent AF. PV reconnection rates differ significantly among AF ablation strategies in patients; after an initial CB procedure, increased PV reconnections compared to RF were documented, and the preferred location of PV reconnection was the RIPV, irrespective of the initial ablation technique. Redo procedures are safe and display long-term success in approximately 70% of patients. The presence of PV reconnection at the time of redo ablation does not impact the long-term outcome. Different re-ablation strategies for patients with and without PV reconnections did not impact the long-term success. Multi-variable predictors for AF recurrence in patients with PV reconnection were female gender, AF/AT as initial rhythm and a higher CHA2DS2 VASc score. 

### 4.2. PV Reconnection Rates during Redo 

In our selected group of patients with recurrences after initial AF ablation, PV reconnection remains a clinical problem despite developments in ablation technologies, such as automated lesion assessment and contact force catheters [13]. During the redo procedure, all PVs were carefully assessed using a circular mapping catheter and proof of entry/exit-block. We found lower numbers of PV reconnections after point-by-point RF-based ablations compared to CB ablations. This finding is not in line with previous studies showing increased lesion durability for CB ablations [6,14]. It is important to keep in mind that the patients included in our analysis were ablated at different centers and do not represent a consecutive series of patients. In the present study of patients with clinically relevant arrhythmia recurrences after AF ablation, the most common finding was PV reconnections. A large proportion of the patients analyzed here were ablated with “older” technologies, including 30 W RF ablations or 1st-generation CB; therefore the results may not be attributable to “newer” ablation strategies [15,16,17]. 

In general, right-sided PVs revealed higher reconnection rates compared to left PVs, irrespective of the RF or CB approach. This finding might be explained by the fact that the catheter approach for right PVs is more challenging and might result in reduced lesion durability. In prior reports, right inferior and left common PVs in particular showed high reconnection rates with cryoballoon and RF; this is supported by our results (although right superior PV also showed high reconnections). With current ablation technologies, the number of PV reconnections during follow-up is expected to reduce; therefore, ablation strategies for redo-ablations may change accordingly.

Patients after surgical AF ablation often present with macro re-entrant tachycardias in addition to reconnected PVs. In our study, 46% of all post-surgical AF ablation patients showed atrial tachycardia at the beginning of the procedure. Previous reports show high PV reconnection rates after surgical AF ablation, with 79–93% of all patients having at least one reconnected PV [18,19]. This is significantly higher than in our series, and might be explained by the different ablation methods used for surgical AF ablation. 

### 4.3. Different Ablation Techniques Used for PVI

Several techniques have emerged for PVI in the last years, most prominently cryoballoon and radiofrequency ablation. These technologies differ in energy source and application mode. RF ablation is applied in a point-by-point mode, resulting in tissue heating and cellular necrosis. Cryoballoon is a single-shot device leading to necrosis via freezing. Each technique has its own advantages, including shorter procedure times and a steeper learning curve [4]. RF procedures, on the other hand, require only the very limited use of fluoroscopy, but demand extensive training of the respective operators [2]. Both techniques have been found comparable in terms of efficacy and safety for paroxysmal AF [4]. In recent years, electroporation using pulse field ablations has emerged and provided promising results regarding procedural safety and efficacy [20]. 

### 4.4. Ablation Strategies for Redo Procedures

PV reconnection might be the main driver of AF recurrence among redo procedures, and PV re-isolation may therefore be the primary goal for every AF redo-ablation [21]. Atrial arrhythmia recurrences remain a predictor of finding PV reconnections in redo procedures, and therefore a strategy towards re-isolating PVs should be available for these cases. Notably, the probability of at least one reconnected PV was highest with sinus rhythm at the beginning of the redo procedure and decreased significantly in the case of macro re-entrant atrial tachycardia. However, in patients without PV reconnections or in patients with LA low-voltage areas, the situation is more complex, and the approach is still not clear. Common protocols include the ablation of non-PV areas empirically or, under the guidance of substrate mapping, the use of linear ablations for atrial compartmentalization, the focal ablation of complex fractionated atrial electrograms (CFAEs) or guided rotor ablations [22]. This is also reflected in significantly longer ablation times within the group without PV reconnection in this study, which might be related to more LA low-voltage areas or when to primarily ablate in the case of isolated PVs being unclear. However, evidence of these procedures is scarce, and possible issues such as atrial dysfunction, conduction abnormalities and iatrogenic macro-re-entrant tachycardias may arise. In our study, the long-term outcomes of patients without PV reconnections were as good as those of patients with PV reconnections undergoing PV re-isolation. We performed different ablation strategies, including the antral extension of prior ablation sets, linear lesion sets, posterior wall isolation and isolation of the SVC. These measures allowed sufficient rhythm control during follow-up in 2/3 of all patients, which is in line with the previous literature [23]. However, recurrence rates of 33% highlight that triggers beyond the PVs are not well understood in this cohort. The “approach of choice” for redo-AF ablation remains unclear. 

Future implications of our study might be that these patients may well be seen as “new AF patients”, to whom the same concepts apply as to an ablation-naive AF patient. Multidisciplinary approaches for those patients with AF recurrence, such as glycemic control and a reduction in body weight, might be even more important and should be added in the treatment plan [24,25].

### 4.5. Complications in AF Ablation Procedures

Recent studies suggest that AF ablation is associated with a similar or even lower risk of adverse cardiovascular outcomes compared to antiarrhythmic therapies, challenging the established use of drugs as an initial therapy [26,27]. Multiple studies show a wide variety of complication rates, ranging from 1 to 8% depending on the type of study and definitions. The most common complications are related to vascular access [28]. Ultrasound-guided vascular access can decrease the risk of these types of complications dramatically and enhance procedural efficacy [29,30]. Fortunately, other severe complications such as pericardial tamponade/effusion or other seldom-severe complications have decreased over time [28]. In summary, a significant improvement in the safety profile of AF ablations can be observed.

### 4.6. Limitations

The observational design is a major limitation of this study. The lost to follow-up rate was 2%. This is a single-center study, and the results of this study may not be transferrable to other centers using different ablation protocols with different catheters or settings.

The patients included were consecutive patients undergoing their first redo AF ablation after prior ablations performed at different centers with different expertise and modalities, although a large number had undergone first ablation in our center. This study included a heterogeneous study population treated with heterogeneous ablation strategies in a non-randomized setting and a retrospective analysis of different ablation approaches. Patients undergoing first PVI during surgery constitute a different population to those treated with an interventional approach, and the presence and rates of PV reconnections should be interpreted carefully. 

## 5. Conclusions

PV reconnections after initial successful PVI are common among all AF ablation techniques, with higher rates among CB-based techniques in our series. Patients with PV reconnection revealed long-term results comparable with patients with electrically silent PVs. Rhythm control was possible in 2/3 of all patients after the redo procedure; however, the use of different ablation strategies with extra-PV trigger ablation did not improve long-term success. Patients with recurrent AF after PVI constitute a challenging group of patients. 

## Figures and Tables

**Table 1:** Baseline characteristics.

Characteristic	All Patients (*n* = 389; 100%)		PV Reconnection (*n* = 325; 84%)		No PV Reconnection (*n* = 64; 16%)		*p* Value
Age, median (range)	68 (22–88)		67 (22–84)		70 (26–88)		**0.021**
Males, *n* (%)	220	(57)	197	(61)	23	(36)	**0.001**
Paroxysmal AF, *n* (%)	150	(39)	132	(41)	18	(28)	0.061
Arterial hypertension, *n* (%)	341	(88)	285	(88)	56	(88)	0.966
Diabetes mellitus, *n* (%)	65	(17)	50	(15)	15	(23)	0.114
CAD, *n* (%)	110	(28)	94	(29)	16	(25)	0.524
History of stroke/TIA, *n* (%)	24	(6)	19	(6)	5	(8)	0.550
LVEF (%)	55 ± 10		55 ± 10		54 ± 12		0.483
LA size (qcm)	26.0 ± 6.9		25.8 ± 6.5		27.1 ± 9.0		0.227
BMI	29.1 ± 6.3		29.0 ± 6.5		28.9 ± 5.1		0.921
CHA2DS2-VASc score	3.0 ± 1.5		2.9 ± 1.4		3.5 ± 1.6		**0.001**

AF, atrial fibrillation; BMI, body mass index; CAD, coronary artery disease; LA, left atrium; LVEF, left ventricular ejection fraction; PV, pulmonary vein; TIA, transient ischemic attack. Bold type indicates statistical significance *p* < 0.05.

**Table 2:** Procedural data.

Characteristic	All Patients (*n* = 389; 100%)		PV Reconnection (*n* = 325; 84%)		No PV Reconnection (*n* = 64; 16%)		*p* Value
Mean number of reconnected PVs, mean ± SD	2.0 ± 1.3		2.4 ± 1.1		0.0 ± 0.0		-
LSPV reconnection	168	(43)	168	(52)	0	(0)	-
LIPV reconnection	134	(35)	134	(41)	0	(0)	-
RSPV reconnection	229	(59)	229	(71)	0	(0)	-
RIPV reconnection	238	(61)	238	(74)	0	(0)	-
Left common trunk reconnection	4	(100)	4	(100)	0	(0)	-
Low voltage overall	227	(58)	164	(51)	63	(98)	**0.001**
Mean number of LVZ, mean ± SD	1.3 ± 1.3		1.0 ± 1.2		2.6 ± 1.2		**0.001**
Low-voltage anterior	169	(44)	120	(37)	49	(77)	**0.001**
Low-voltage roof	125	(32)	84	(26)	41	(64)	**0.001**
Low-voltage posterior	136	(35)	90	(28)	46	(72)	**0.001**
Low-voltage septal	62	(16)	33	(10)	29	(45)	**0.001**
Roof line	99	(26)	71	(22)	28	(44)	**0.001**
Mitral line	148	(38)	104	(32)	44	(69)	**0.001**
Box lesion	111	(29)	77	(24)	34	(53)	**0.001**
SVC	181	(47)	150	(46)	31	(48)	0.754
Septal line	30	(8)	17	(5)	13	(20)	**0.001**
CTI	39	(10)	27	(8)	12	(19)	**0.011**
Procedural duration (min), mean ± SD	87.5 ± 27.2		87.0 ± 27.6		90.0 ± 24.7		0.479
Fluoroscopy duration (min), mean ± SD	7.6 ± 5.2		7.5 ± 5.3		7.9 ± 4.3		0.595
Ablation duration (min), mean ± SD	13.4 ± 8.4		12.9 ± 8.3		15.6 ± 9.3		**0.023**

CTI, cavotrikuspidal isthmus; LSPV, left superior pulmonary vein; LIPV, left inferior pulmonary vein; LVZ, low voltage zones; RSPV, right superior pulmonary vein; RIPV, right inferior pulmonary vein; PV, pulmonary vein; SD, standard deviation; SVC, superior vena cava. Bold type indicates statistical significance *p* < 0.05.

**Table 3:** PV reconnections stratified according to ablation technique.

Characteristic	RF 30–35 W (*n* = 178; 46%)		RF 50 W (*n* = 96; 25%)		RF 90 W (*n* = 4; 1%)		CB 1st Gen (*n* = 31; 8%)		CB 2nd Gen (*n* = 17; 4%)		CB 3rd Gen (*n* = 37; 10%)		Surgical (*n* = 26; 7%)		*p* Value
LSPV, *n* (%)	72	(40)	36	(38)	1	(25)	20	(65)	9	(53)	20	(54)	10	(39)	0.102
LIPV, *n* (%)	54	(30)	30	(32)	2	(50)	16	(52)	10	(59)	15	(41)	7	(27)	0.065
RSPV, *n* (%)	110	(62)	51	(54)	4	(100)	21	(68)	12	(71)	24	(65)	7	(27)	**0.006**
RIPV, *n* (%)	111	(62)	58	(61)	3	(75)	22	(71)	11	(65)	25	(68)	8	(31)	**0.051**
At least one PV reconnected, *n* (%)	151	(85)	76	(79)	4	(100)	31	(100)	16	(94)	34	(92)	13	(50)	**0.001**
Mean number of reconnected PVs, mean ± SD	1.9 ± 1.2		1.8 ± 1.3		2.5 ± 0.6		2.5 ± 1.0		2.5 ± 1.5		2.3 ± 1.3		1.2 ± 1.6		**0.002**
Mean time between first and redo, mean ± SD (months)	42 ± 30		11 ± 6		9 ± 4		122 ± 40		62 ± 24		16 ± 11		48 ± 44		**0.001**

CB, cryoballoon; LSPV, left superior pulmonary vein; LIPV, left inferior pulmonary vein; PV, pulmonary vein; RF, radiofrequency; RSPV, right superior pulmonary vein; RIPV, right inferior pulmonary vein; SD, standard deviation. Bold type indicates statistical significance *p* < 0.05.

**Table 4:** Endpoints.

Characteristic	All Patients (*n* = 389; 100%)		PV Reconnection (*n* = 325; 84%)		No PV Reconnection (*n* = 64; 16%)		*p* Value
Intraprocedural PV reconnection	18	(5)	18	(6)	0	(0)	**0.053**
Intrahospital AF recurrence	38	(10)	34	(11)	4	(6)	0.297
Sinus rhythm at 3 months	332	(87)	275	(86)	57	(89)	0.539
Sinus rhythm at 12 months	277	(73)	229	(72)	48	(75)	0.625
Sinus rhythm at end of follow-up	255	(67)	212	(67)	43	(67)	0.936

AF, atrial fibrillation; PV, pulmonary vein. Bold type indicates statistical significance *p* < 0.05.

**Table 5:** The uni- and multi-variable hazard ratios used to predict recurrence of atrial tachycardia after follow-up in all patients (*n* = 389).

	Uni-Variable			Multi-Variable		
	HR	95% CI	*p* Value	HR	95% CI	*p* Value
Age	1.020	1.001–1.041	**0.044**	-	-	-
BMI	1.020	0.993–1.049	0.152	-	-	-
Number of reconnected PVs	0.918	0.806–1.046	0.200	-	-	-
Persistent AF	1.203	0.838–1.727	0.316	-	-	-
LA size	1.016	0.992–1.041	0.200	-	-	-
CHA2DS2 VASc Score	1.206	1.079–1.349	**0.001**	1.281	1.027–1.598	**0.028**
Rhythm at start of redo	1.171	0.947–1.448	0.145	-	-	-
Female gender	1.857	1.195–2.381	**0.018**	2.145	1.725–3.412	**0.007**
Number of LVA	1.113	0.982–1.261	0.094	-	-	-
Ablation strategy	1.072	1.001–1.147	**0.046**	-	-	-

AF, atrial fibrillation; BMI, body mass index; CI, confidence interval; HR, hazard ratio; LA, left atrium; LVA, low voltage area; PV, pulmonary vein. Bold type indicates statistical significance *p* < 0.05.

**Table 6:** The uni- and multi-variable hazard ratios used to predict recurrence of atrial tachycardia after follow-up in patients with PV reconnection and without LA LVA (*n* = 166).

	Uni-Variable			Multi-Variable		
	HR	95% CI	*p* Value	HR	95% CI	*p* Value
Age	1.023	0.994–1.055	0.144	-	-	-
BMI	1.031	0.989–1.074	0.147	-	-	-
Number of reconnected PVs	0.862	0.656–1.133	0.287	-	-	-
Persistent AF	1.410	0.806–2.467	0.229	-	-	-
LA size	1.013	0.965–1.063	0.603	-	-	-
CHA2D2S2 VASc Score	1.426	1.176–1.729	**0.001**	1.281	1.027–1.598	**0.028**
Rhythm at start of redo	1.380	0.917–2.077	0.098	1.656	1.598–2.604	**0.029**
Female gender	1.943	1.943–3.476	**0.001**	2.108	1.615–4.012	**0.007**
Ablation strategy	1.362	1.012–1.834	**0.042**	-	-	-

AF, atrial fibrillation; BMI, body mass index; CI, confidence interval; HR, hazard ratio; LA, left atrium; PV, pulmonary vein. Bold type indicates statistical significance *p* < 0.05.

**Table 7:** The uni- and multi-variable hazard ratios used to predict recurrence of atrial tachycardia after follow-up in patients with PV reconnection and LA LVA (*n* = 230).

	Uni-Variable			Multi-Variable		
	HR	95% CI	*p* Value	HR	95% CI	*p* Value
Age	1.015	0.982–1.050	0.379	-	-	-
BMI	1.022	0.980–1.065	0.318	-	-	-
Number of reconnected PVs	0.869	0.681–1.108	0.257	-	-	-
Persistent AF	1.092	0.614–1.941	0.765	-	-	-
LA size	1.037	0.999–1.077	0.059	-	-	-
CHA2DS2 VASc Score	1.099	0.918–1.316	0.303	-	-	-
Rhythm at start of redo	1.105	0.813–1.502	0.524	-	-	-
Female gender	0.936	0.562–1.560	0.801	-	-	-
Number of LVA	1.066	0.823–1.381	0.626	-	-	-
Ablation strategy	1.087	0.924–1.225	0.174	-	-	-

AF, atrial fibrillation; BMI, body mass index; CI, confidence interval; HR, hazard ratio; LA, left atrium; LVA, low voltage are; PV, pulmonary vein.

## Data Availability

Data is available from the corresponding author upon reasonable request.
